# Cortical excitability and its modulation in obsessive-compulsive disorder - a systematic review

**DOI:** 10.1192/j.eurpsy.2021.1311

**Published:** 2021-08-13

**Authors:** D. Silva, A. Maia, G. Cotovio, J. Oliveira, A. Oliveira-Maia, B. Barahona-Correa

**Affiliations:** 1 Neuropsychiatry Unit, Champalimaud Center for the unkonw, Lisboa, Portugal; 2 Neurospychiatry Unit, Centro Clínico Champalimaud, Lisboa, Portugal; 3 Neuropsychiatry Unit, Champalimaud Research and Clinical Centre, Champalimaud Foundation Centre for the Unknown, Lisbon, Portugal

**Keywords:** Obsessive-Compulsive disorder, transcranial magnetic stimulation, Neurophysiology, Cortical excitability

## Abstract

**Introduction:**

Obsessive-Compulsive Disorder (OCD) is an incapacitating Neuropsychiatric condition characterized by the presence of obsessions and/or compulsions. Although the disorder’s phenotype is well described, its pathophysiology remains elusive (Aouizerate et al, 2004). Over the last decade, techniques to noninvasively study the brain’s neurophysiology, such as Transcranial Magnetic Stimulation (TMS), have found widespread use in psychiatric research. For OCD, single- and paired-pule TMS protocols have been used to explore abnormalities in motor cortex excitability and cortical neuroplasticity. Here we propose to systematically review and, where possible, metanalyse existing case-control studies that compared such measures in patients and healthy subjects.

**Objectives:**

To systematically review and meta-analyse published case-control studies comparing cortical excitability measures, as measured by single- or paired-pulse TMS, in subjects with OCD and healthy controls.

**Methods:**

We have conducted a systematic review of published literature (PROSPERO registration CRD42020201764) reporting measures of cortical excitability as measured by single or paired-pulse TMS, in patients with OCD and healthy controls. We searched 4 different electronic libraries (PubMed, Web of Science, EMBASE, PsycINFO). The resulting list of articles was reviewed, separately, by two researchers. Disagreements were discussed and resolved by consensus, until a final list of eligible articles was obtained.

**Results:**

13 studies reporting motor cortex excitability measures were included in our final list. The total number of participants included in our analyses is 615 (349 OCD; 180 healthy subjects; 86 other conditions)

**Conclusions:**

A sufficient number of studies was found to allow for metanalyses, currently ongoing.

